# A study on collaboration innovation: Perspectives of innovative ecosystems of value co-creation using evolutionary game theory

**DOI:** 10.1371/journal.pone.0339295

**Published:** 2026-02-11

**Authors:** Xiaowei Shi, Jifa Wang

**Affiliations:** School of management, Shenyang University of Technology, Shenyang, Liaoning Province, China; Covenant University, NIGERIA

## Abstract

Enhancing emphasis on value co-creation and optimizing resource allocation are crucial for fostering deep coupling and synergistic development within innovation ecosystems, thereby enhancing innovation efficacy. Within a bounded rationality framework, this study constructs an evolutionary game model of value co-creation behavior between core enterprises and participating firms in innovation ecosystems. Simulation analysis reveals the underlying evolutionary dynamics. Findings indicate that sufficient resource reserves, superior value creation capabilities, positive cognitive attitudes, effective incentive policies, and appropriate regulatory measures all facilitate high-quality value co-creation. Conversely, heightened uncertainty risks and substantial co-creation costs may impede value co-creation among innovation actors within the ecosystem. The results hold significant implications for optimizing value co-creation mechanisms and promoting the healthy development of innovation ecosystems.

## 1. Introduction

In the context of open innovation, it is difficult for individual firms to maintain a significant competitive advantage in technological innovation competition [1]. Within an innovation ecosystem, however, various innovators reach agreements through contracts concerning production decisions, revenue distribution, and dispute resolution. By fulfilling contractual obligations and investing resources, they engage in collaborative innovation to achieve value co-creation [2]. This approach not only fosters open collaboration among innovators and facilitates resource sharing but also emphasizes dynamic interdependencies and interactions, thereby addressing the limitation that individual firms often cannot independently meet all resource requirements during technological innovation [3]. In such ecosystems, core enterprises serve as foundational pillars, playing a critical role in resource aggregation. Leveraging their dominant position, they drive the clustering and development of participating entities [4]. Concurrently, participating firms must recognize and accept the leadership of core enterprises, aligning closely with them in terms of vision for value co-creation, governance mechanisms, and operational guidelines [5]. While traditional innovation strategies focus on enhancing a single firm’s competitive edge, innovation ecosystem strategies prioritize managing inter-firm synergies to realize collective value creation. This shift not only enhances the overall competitiveness of the entire ecosystem but also reinforces the central role of core enterprises within it [6]. Therefore, to strengthen the competitive advantages of innovation ecosystems, core enterprises must effectively allocate and integrate systemic innovation resources, elevate the level of value co-creation across the network, and thereby advance the sustainable development and enhanced competitiveness of the entire ecosystem [7].

Nevertheless, innovation ecosystems may encounter numerous challenges during the process of value co-creation [8], including resource scarcity and misuse, information opacity, goal divergence, as well as other inappropriate or destructive behaviors. These issues can lead to suboptimal quality of value outcomes, even negative impacts, ultimately causing the system to deviate from its original intent of value co-creation and veer toward its antithesis—value co-destruction [9]. Within such ecosystems, information asymmetry among actors often fosters opportunistic conduct, with “free-riding” being a particularly salient example [10]. Due to disparities in patent information, some firms might exploit their secondary patents to masquerade as high-value assets, seizing advantageous resources from the shared pool without genuinely contributing to collaborative innovation and value co-creation. It is precisely these disruptive actions that prompt other innovators to exercise caution or outright refuse to share their core technological knowledge, thereby triggering a vicious cycle. Moreover, this erosion of trust and constriction of resource flows may ultimately precipitate ecosystem failure or even collapse [8]. Therefore, to prevent innovation ecosystems from descending into value co-destruction, enhance innovation efficacy, and promote mutual economic and ecological advancement, it is imperative to intensify focus on value co-creation, establish robust information-sharing mechanisms, optimize resource allocation, and strengthen oversight against opportunistic behaviors. Only thus can all entities within the system authentically engage in collaborative innovation and collectively advance value creation.

Scholars posit that value co-creation in innovation ecosystems constitutes a macro-level phenomenon requiring meso-level analysis, emphasizing open collaboration, interactive dynamics, and resource sharing among ecosystem entities [11]. Firstly, open collaboration within innovation ecosystems not only continuously generates innovative opportunities but also enhances overall systemic value [12]. Secondly, through open innovation and resource sharing, members achieve value co-creation [3], manifesting both in fulfilling individual entity needs and generating collective benefits across all participants during service innovation processes, thereby establishing virtuous cycles [13]. Current research on value co-creation in innovation ecosystems primarily focuses on two domains: First, coordination mechanisms for value co-creation: Dai et al demonstrates that heterogeneity in roles and resource endowments enables entities to satisfy individual value acquisition needs through complementary advantages while advancing ecosystem objectives, thus facilitating value co-creation [14]. Hilbolling et al. examining interdependent value co-creation among platform owners, complementors, and users in Philips’ innovation ecosystem, empirically validated that multi-agent cooperation significantly promotes ecosystem-wide value co-creation [15]. Bonina et al. found that by providing diverse functionalities and interfaces enabling broad generation of varied innovations, ecosystems grant participants access to diversified resources conducive to value co-creation [16]. Second, governance drivers of value co-creation: Wulf et al. identified knowledge-sharing mechanisms between partners as critical enablers, enhancing collaborative efficiency, optimizing resource allocation, and strengthening ecosystem innovation capacity [17]. Valkokari et al. revealed that greater diversity among participants amplifies support for innovation within value co-creation processes [18]. Dong et al. positioned core leadership and complementor performance as central elements in digital innovation ecosystem value co-creation, with interconnected factors driving high-value outcomes [19]. Wang et al. empirical study identified multiple determinants influencing participant behavior: satisfaction with ecosystem services/technological breakthroughs, ecosystem value orientation, resource-sharing institutions, trust in lead firms, and environmental drivers [20]. Furthermore, evolutionary game perspectives illuminate key factors shaping co-creation dynamics: the distribution ratio of excess value co-creation gains, coordination costs, payoffs from deceptive strategies, returns from unilateral cooperation, and efficacy of reward-penalty systems [21]. Jiang et al. proposed exploring value co-creation mechanisms through ecosystem framework construction and firm-level ecological niche strategic decision-making [22]. Wang et al., applying evolutionary game theory, demonstrated that process emission levels, environmental quality improvements, and inter-organizational payment structures significantly influence the entire value co-creation trajectory [23].

Although scholars have examined value co-creation behavior in innovation ecosystems from a game-theoretic perspective and derived relevant conclusions [21], value creation and value capture represent two critical stages of value co-creation among actors. Value creation focuses on the process by which entities interact collaboratively to generate value at the ecosystem level, while value capture emphasizes how individual firms acquire benefits and establish competitive advantages at the firm level [24]. Therefore, grounded in value co-creation theory and assuming bounded rationality among strategic players, this study analyzes the cost-benefit tradeoffs inherent in value co-creation between core enterprises and participating firms within innovation ecosystems. Based on existing research and practical requirements, we select key factors—including resource endowment, value co-creation efficacy, cognitive orientation toward co-creation, incentive mechanisms, punitive measures, associated risks, and implementation costs—to construct an evolutionary game model of value co-creation behavior. Through numerical simulations, we derive conclusions and offer recommendations. This approach partially addresses gaps in current literature concerning the optimization of value creation and capture strategies across diverse innovation actors within ecosystems. It extends theoretical understanding of determinants influencing value co-creation in innovation ecosystems. Additionally, our findings provide theoretical foundations and practical insights for optimizing value creation/capture decisions, facilitating effective value co-creation, enhancing performance outcomes in innovation ecosystem practice, and ultimately promoting sustainable co-evolution within these systems.

## 2. Model construction

Evolutionary game theory integrates principles from evolutionary biology and classical game theory, positing that boundedly rational players continuously adjust their strategies in response to environmental dynamics to pursue optimality and ultimately reach dynamic equilibrium states [25]. Within innovation ecosystems, participants cannot instantaneously identify optimal strategies; instead, they engage in iterative learning, adaptation, and repeated games to maximize individual interests and requirements—a process epitomizing the core tenets of evolutionary game thinking [26]. In innovation ecosystem research, Zou et al. employed evolutionary game theory to construct a model centered on core enterprises as leaders with upstream/downstream firms as participants, investigating determinants influencing inter-firm collaborative cooperation [27]. Yang utilized an evolutionary game framework to explore key factors stabilizing multi-agent collaborative innovation within ecosystems [28]. Regarding value co-creation specifically, scholars [4,23] have initiated relevant investigations. Given that evolutionary game simulation models effectively analyze strategic decision-making during long-term repeated interactions among multiple actors in innovation ecosystems, this study draws upon value co-creation theory and leverages evolutionary game modeling to examine factors shaping choices between value creation and capture made by core enterprises and participating firms throughout the value co-creation process.

### 2.1. Variable description

This study synthesizes prior research to identify determinants spanning the dual dimensions of value creation and capture within innovation ecosystem co-creation dynamics. Key factors encompass: resource endowment, co-creation efficacy coefficient, cognitive orientation toward collaboration, incentive intensity for joint value generation, punitive measures against defection, inherent risk exposure during collaborative processes, and implementation costs associated with coordinated efforts. Detailed specifications follow:

#### 2.1.1. Resource quantity (Q).

Resource quantity primarily refers to the total ownership of such resources by leading firms or participant enterprises during value creation, rather than the amount expended in the process. This distinction exists because these entities only consume a portion of their owned resources during value creation, implying a certain conversion rate in their resource utilization for value generation.

#### 2.1.2. Co-creation efficiency coefficient.

The co-creation efficiency coefficient here encompasses two aspects: the value creation coefficient (α) and the value capture coefficient (β), and 0≤α,β≤1. Both value creation and value capture involve a certain conversion rate. This paper posits that value capture is conducted based on the foundation of value creation.

#### 2.1.3. Co-creation awareness coefficient (γ).

If the co-creation awareness coefficient of leading firms or participant enterprises is higher, the probability of their engaging in value creation increases, and such value creation behavior becomes more significant. Here, 0≪γ≪1, with co-creation awareness serving as the foundational basis for value creation.

#### 2.1.4. Co-creation reward coefficient (δ).

Rewards are implemented for leading firms or participant enterprises engaged in value creation. A higher reward coefficient indicates greater value created by these entities, making their value creation behavior more significant, and 0≤δ≤1. Co-creation rewards primarily involve the transfer of resources derived from value creation by leading firms or participants, rather than representing the total ownership of resources.

#### 2.1.5. Co-creation penalty coefficient (ε).

This coefficient targets behaviors that do not engage in value creation but solely capture value, implementing corresponding penalty measures. Similar to rewards, a larger penalty coefficient amplifies the significance of value creation behavior, with 0≤ε≤1. Co-creation penalties should be based on the amount of resources transferred during value creation, rather than the total ownership of resources.

#### 2.1.6. Co-creation risk coefficient (θ).

Leading firms and participant enterprises face inherent risks during value creation, such as misalignment between value creation and value capture, and the risk of diminished autonomous competitiveness due to system-dependent value creation. The co-creation risk is directly proportional to the resource conversion rate in the value creation process, and 0≤θ≤1.

#### 2.1.7. Co-creation cost coefficient (ϕ).

Co-creation costs primarily refer to the expenses arising from the conversion of resources during the value creation process. This paper therefore posits that co-creation costs are directly proportional to the resource conversion rate in value creation, and 0≤ϕ≤1.

### 2.2. Model assumptions

Core enterprises occupy central positions within innovation ecosystems, guiding systemic development and exerting significant influence over technological innovation, network coordination, and ecosystem evolution [29]. Non-core participants follow lead firms’ trajectories through technological imitation, complementation, or substitution—establishing niche differentiation that enhances scale effects and synergistic interactions across the ecosystem while advancing collective innovation upgrading [30]. The dynamic competitive-collaborative relationships between core and participating entities form foundational mechanisms for sustainable ecosystem development. A perspective grounded in differentiated population ecology offers novel insights into strategic choices among core enterprises [31]. Recognizing that all actors continuously seek equilibrium between value creation and capture [32]—a perpetually adjusted process exhibiting characteristics of ecological population dynamics. This study dissects from a dynamic viewpoint the endogenous mechanisms governing strategy selection regarding value creation and appropriation by both core and participant firms during self-organizing ecosystem evolution. We analyze formation pathways and evolutionary patterns of these dual processes accordingly, leading to the following hypotheses:

Assumption 1: Both the leading firm and participating enterprises choose the strategy of value creation.

In this scenario, the payoffs for both parties are composed of the following components: Value gains accruing to the leading firm from the value created by participating entities; Value derived from co-creation awareness for the leading firm; Rewards received by the leading firm for engaging in value creation; Risks associated with value creation; Costs incurred by the leading firm in creating value. The total payoff for the leading firm in this process is $\beta_{m}\alpha_{n}Q_{n}+\gamma \alpha_{m}Q_{m}+\delta \alpha_{m}Q_{m}-\theta Q_{m}-\varphi Q_{m}$, while that for the participating enterprises is $\beta_{n}\alpha_{m}Q_{m}+\gamma \alpha_{n}Q_{n}+\delta \alpha_{n}Q_{n}-\theta Q_{n}-\varphi Q_{n}$.

Assumption 2: The leading firm and participating enterprises adopt opposite strategies—one chooses value creation while the other opts for value capture.

When the leading enterprise creates value while the participating enterprise merely captures it, the revenue obtained by the value-creating party (assumed to be the leading enterprise) primarily comprises: benefits generated from co-created awareness, rewards from value creation, risks associated with value creation, and costs incurred for value creation. Therefore, the total revenue of the leading enterprise throughout this process is: $\gamma \alpha_{m}Q_{m}+\delta \alpha_{m}Q_{m}-\theta Q_{m}-\varphi Q_{m}$. As for the party acquiring value (assumed to be the participating enterprises), their total revenue comprises two components: the benefits derived from capturing the value created by the leading enterprise, and the penalties incurred solely for engaging in value acquisition. Consequently, the total revenue of the participating enterprises throughout this process amounts to: $\beta_{n}\alpha_{m}Q_{m}-\varepsilon \alpha_{m}Q_{m}$.

Assumption 3: Both the leading enterprise and the participating enterprises choose a value-capturing strategy.

When both enterprises focus exclusively on value capture, the total payoffs for both the leading enterprise and the participating enterprises is 0.

### 2.3. Payoff matrix

Based on the above assumptions, a benefit analysis is conducted from the perspectives of revenue, cost, and risk, leading to the construction of a payoff matrix for value co-creation behavior decisions between leading firms and participating enterprises in an innovation ecosystem, as shown in Table 1. It is assumed that the probability of a leading firm choosing value creation is X, with the probability of value capture being 1−X; similarly, for participating enterprises, the probability of value creation is Y, and the probability of value capture is 1−Y, where 0≤X,Y≤1.

**Table 1:** Payoff matrix of leading enterprise m and participating enterprise n.

		Participating Enterprise (n)	
		Value creation (Y)	Value capture (1−Y)
Leading Enterprise (m)	Value creation(X)	$\beta_{m}\alpha_{n}Q_{n}+\gamma \alpha_{m}Q_{m}+\delta \alpha_{m}Q_{m}-\theta Q_{m}-\varphi Q_{m}$ $\beta_{n}\alpha_{m}Q_{m}+\gamma \alpha_{n}Q_{n}+\delta \alpha_{n}Q_{n}-\theta Q_{n}-\varphi Q_{n}$	$\gamma \alpha_{m}Q_{m}+\delta \alpha_{m}Q_{m}-\theta Q_{m}-\varphi Q_{m}$ $\beta_{n}\alpha_{m}Q_{m}-\varepsilon \alpha_{m}Q_{m}$
	Value capture(1−X)	$\beta_{m}\alpha_{n}Q_{n}-\varepsilon \alpha_{n}Q_{n}$ $\gamma \alpha_{n}Q_{n}+\delta \alpha_{n}Q_{n}-\theta Q_{n}-\varphi Q_{n}$	*0* *0*

### 2.4. Solution of the evolutionary game model

Before establishing the evolutionary game system and constructing the evolutionary game model, define the expected returns of value creation for the leading enterprise and participating enterprises in the innovation ecosystem as $E_{\alpha }(m)$ and $E_{\alpha }(n)$ respectively, the expected returns of value capture for the leading enterprise and participating enterprises as $E_{\beta }(m)$ and $E_{\beta }(n)$ respectively, and the overall expected returns for the leading enterprise and participating enterprises as E(m) and E(n) respectively.

The expected returns of the leading enterprise m during the value creation process is: $E_{\alpha }(m)=Y(\beta_{m}\alpha_{n}Q_{n}+\gamma \alpha_{m}Q_{m}+\delta \alpha_{m}Q_{m}-\theta Q_{m}-\varphi Q_{m})+(1-Y)(\gamma \alpha_{m}Q_{m}+\delta \alpha_{m}Q_{m}-\theta Q_{m}-\varphi Q_{m})$. The expected returns of the leading enterprise m during the value capture process is: $E_{\beta }(m)=Y(\beta_{m}\alpha_{n}Q_{n}-\varepsilon \alpha_{n}Q_{n})$. The average expected returns of the leading enterprise m is: $E(m)=XE_{\alpha }(m)+(1-X)E_{\beta }(m)$.

Therefore, the replication dynamic equation of the leading enterprise m is:


$$F(X)=\frac{d_{X}}{d_{t}}=X[E_{\alpha }(m)-E(m)]=X(1-X)(Y\varepsilon \alpha_{n}Q_{n}+\gamma \alpha_{m}Q_{m}+\delta \alpha_{m}Q_{m}-\theta Q_{m}-\varphi Q_{m})$$ (1)

And, the replication dynamic equation of the participating enterprise n is:


$$F(Y)=\frac{d_{Y}}{d_{t}}=Y[E_{\alpha }(n)-E(n)]=Y(1-Y)(X\varepsilon \alpha_{m}Q_{m}+\gamma \alpha_{n}Q_{n}+\delta \alpha_{n}Q_{n}-\theta Q_{n}-\varphi Q_{n})$$ (2)

Therefore, the game evolution process of value co-creation behaviors between the leading enterprise and participating enterprises can be represented by the dynamic differential equation system (1) and (2). By setting F(X)=0 and F(Y)=0, we can determine the five local equilibrium points of the evolutionary game system studied in this paper on the plane. The five local equilibrium points of the evolutionary game for the system studied in this paper on plane O(0,0),P(0,1),W(1,0),V(1,1) can be determined: O(0,0),P(0,1),W(1,0),V(1,1), and $G(\frac{\theta Q_{n}+\varphi Q_{n}-\gamma \alpha_{n}Q_{n}-\delta \alpha_{n}Q_{n}}{\varepsilon \alpha_{m}Q_{m}},\frac{\theta Q_{m}+\varphi Q_{m}-\gamma \alpha_{m}Q_{m}-\delta \alpha_{m}Q_{m}}{\varepsilon \alpha_{n}Q_{n}})$. Additionally, from the replication dynamic equations of the leading enterprise and participating enterprises, we can derive:


$$\frac{dF(X)}{dX}=(1-2X)(Y\varepsilon \alpha_{n}Q_{n}+\gamma \alpha_{m}Q_{m}+\delta \alpha_{m}Q_{m}-\theta Q_{m}-\varphi Q_{m})$$ (3)

$$\frac{dF(X)}{dY}=X(1-X)\varepsilon \alpha_{n}Q_{n}$$ (4)

$$\frac{dF(Y)}{dY}=(1-2Y)(X\varepsilon \alpha_{m}Q_{m}+\gamma \alpha_{n}Q_{n}+\delta \alpha_{n}Q_{n}-\theta Q_{n}-\varphi Q_{n})$$ (5)

$$\frac{dF(Y)}{dX}=Y(1-Y)\varepsilon \alpha_{m}Q_{m}$$ (6)

Based on the method proposed by Friedman, which analyzes the local stability of equilibrium points in evolutionary systems through the Jacobian matrix of the game system, this paper derives the Jacobian matrix using Equations (3), (4), (5), and (6) as follows:


$$\begin{array}{c} \left|J\right|=(1-2X)(Y\varepsilon \alpha_{n}Q_{n}+\gamma \alpha_{m}Q_{m}+\delta \alpha_{m}Q_{m}-\theta Q_{m}-\varphi Q_{m})*(1-2Y)(X\varepsilon \alpha_{m}Q_{m}+\gamma \alpha_{n}Q_{n}+\delta \alpha_{n}Q_{n}-\theta Q_{n}-\varphi Q_{n})\\ +X(1-X)\varepsilon \alpha_{n}Q_{n}*Y(1-Y)\varepsilon \alpha_{m}Q_{m} \end{array}$$

The trace trJ of the matrix determinant is:


$$trJ=(1-2X)(Y\varepsilon \alpha_{n}Q_{n}+\gamma \alpha_{m}Q_{m}+\delta \alpha_{m}Q_{m}-\theta Q_{m}-\varphi Q_{m})+(1-2Y)(X\varepsilon \alpha_{m}Q_{m}+\gamma \alpha_{n}Q_{n}+\delta \alpha_{n}Q_{n}-\theta Q_{n}-\varphi Q_{n})$$

Based on the method of analyzing the stability of equilibrium points in evolutionary systems through the local stability of the Jacobian matrix of the game system, it can be concluded that:

① When Model $\varepsilon \alpha_{n}Q_{n}+\gamma \alpha_{m}Q_{m}+\delta \alpha_{m}Q_{m}>\theta Q_{m}+\varphi Q_{m},\varepsilon \alpha_{m}Q_{m}+\gamma \alpha_{n}Q_{n}+\delta \alpha_{n}Q_{n}>\theta Q_{n}+\varphi Q_{n}$ holds, i.e., in the innovation ecosystem, the leading enterprise and participating enterprises engage in value co-creation. If the benefits obtained from value creation simultaneously exceed the sum of their respective costs and the benefits from solely engaging in value capture, the system exhibits five equilibrium points. Among these, O(0,0) and V(1,1) are evolutionarily stable strategy (ESS) points, P(0,1) and W(1,0) are unstable points, $G(\frac{\theta Q_{n}+\varphi Q_{n}-\gamma \alpha_{n}Q_{n}-\delta \alpha_{n}Q_{n}}{\varepsilon \alpha_{m}Q_{m}},\frac{\theta Q_{m}+\varphi Q_{m}-\gamma \alpha_{m}Q_{m}-\delta \alpha_{m}Q_{m}}{\varepsilon \alpha_{n}Q_{n}})$ is a saddle point, and the evolutionary stability results of each equilibrium point are shown in Table 2.

**Table 2:** Local stability of the system when $\varepsilon \alpha_{n}Q_{n}+\gamma \alpha_{m}Q_{m}+\delta \alpha_{m}Q_{m}>\theta Q_{m}+\varphi Q_{m},\varepsilon \alpha_{m}Q_{m}+\gamma \alpha_{n}Q_{n}+\delta \alpha_{n}Q_{n}>\theta Q_{n}+\varphi Q_{n}$.

Equilibrium point	Sign of |J|	Sign of trJ	Local stability
O (0,0)	+	–	ESS
P (0,1)	+	+	Unstable
W (1,0)	+	+	Unstable
V (1,1)	+	–	ESS
$G(\frac{\theta Q_{n}+\varphi Q_{n}-\gamma \alpha_{n}Q_{n}-\delta \alpha_{n}Q_{n}}{\varepsilon \alpha_{m}Q_{m}},\frac{\theta Q_{m}+\varphi Q_{m}-\gamma \alpha_{m}Q_{m}-\delta \alpha_{m}Q_{m}}{\varepsilon \alpha_{n}Q_{n}})$	–	0	Saddle point

**Fig 1:**
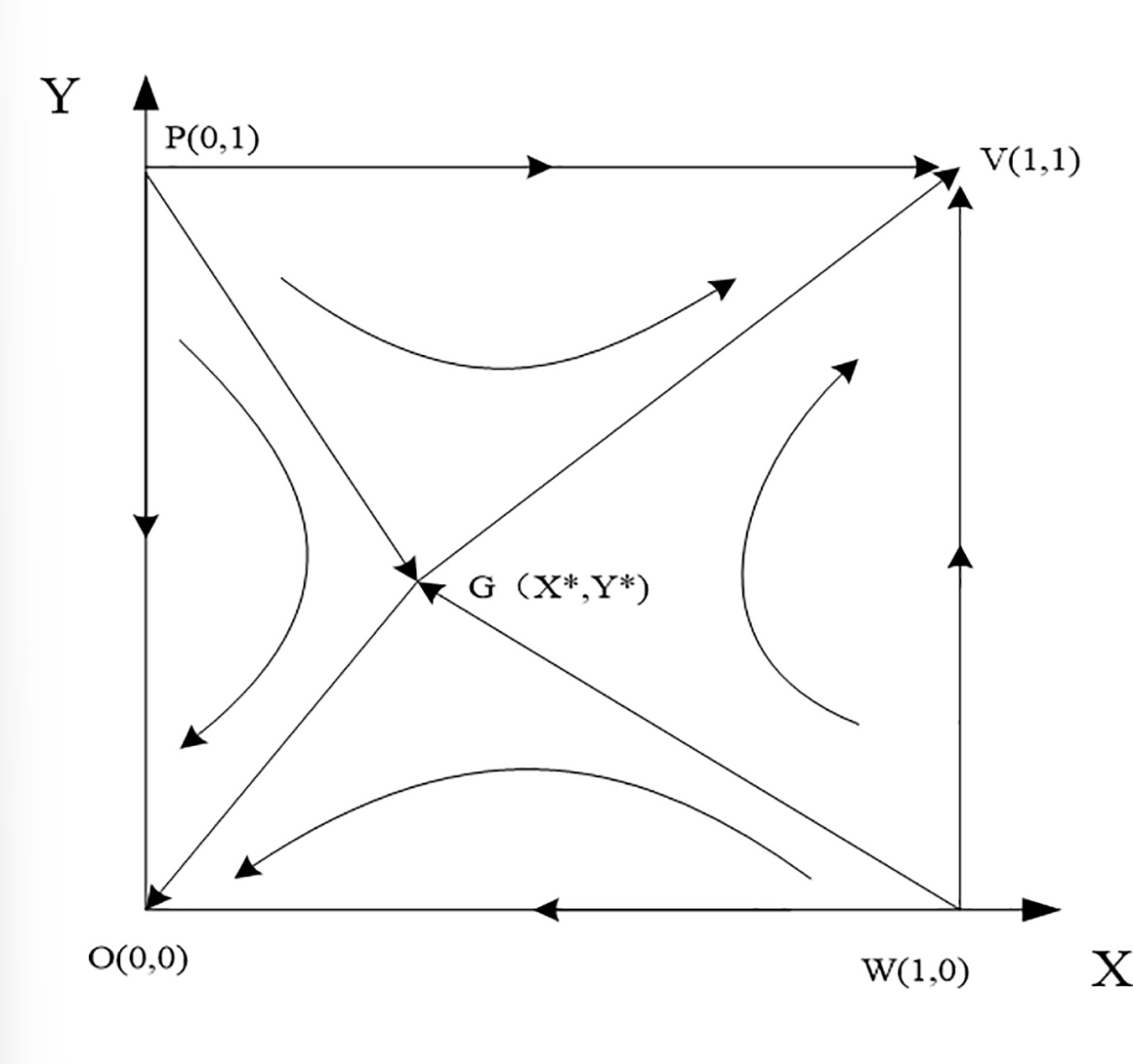
Value co-creation dynamic evolution framework when $\varepsilon \alpha_{n}Q_{n}+\gamma \alpha_{m}Q_{m}+\delta \alpha_{m}Q_{m}>\theta Q_{m}+\varphi Q_{m},\varepsilon \alpha_{m}Q_{m}+\gamma \alpha_{n}Q_{n}+\delta \alpha_{n}Q_{n}>\theta Q_{n}+\varphi Q_{n}$.

Now, using the phase diagram in Fig 1, we represent the dynamic evolution trajectories of the leading enterprise and the participating enterprises. As shown in Fig 1, the two unstable equilibrium points P and W, along with the saddle point G, form a broken line P−G−W, which serves as the critical line for the game parties under different states. When the initial state of the leading enterprise and the participating enterprises is in the lower-left region (OPGW) below the broken line, the system will converge to O(0,0), where both the leading enterprise and the participating enterprises will choose value-capturing strategies; when the initial state of the leading enterprise and the participating enterprises is in the upper-right region (VPGW) above the broken line, the system will converge to V(1,1), where both the leading enterprise and the participating enterprises will choose value-creating strategies.

② When Model $\varepsilon \alpha_{n}Q_{n}+\gamma \alpha_{m}Q_{m}+\delta \alpha_{m}Q_{m}>\theta Q_{m}+\varphi Q_{m},\varepsilon \alpha_{m}Q_{m}+\gamma \alpha_{n}Q_{n}+\delta \alpha_{n}Q_{n}>\theta Q_{n}+\varphi Q_{n}$ does not hold. That is, in the innovation ecosystem, when the combined benefits obtained by the anchor firm and participating enterprises from value co-creation fail to exceed the sum of their respective costs and the gains from mere value capture, the local stability analysis of the system’s Jacobian matrix reveals that the model possesses only one evolutionary stable equilibrium point, O(0,0). This implies that, regardless of their initial states, both the anchor firm and participating enterprises will converge to a strategy of exclusive value capture as the evolutionarily stable strategy (ESS) during the value co-creation process.

## 3. Analysis of evolutionary game model

The evolutionary game model reveals that the evolutionarily stable strategy (ESS) of the dynamic game between the anchor firm and participating enterprises ultimately converges to either both parties adopting value creation strategies or both opting for value capture strategies. The system’s evolutionary trajectory depends primarily on the initial state of the payoff matrix and the direction of evolution, while the final convergence point is also shaped by other interacting factors. By analyzing the phase diagram in Fig 1, the area $S_{VPGW}$ of the VPGW region can be calculated. Given assumption $\varepsilon \alpha_{n}Q_{n}+\gamma \alpha_{m}Q_{m}+\delta \alpha_{m}Q_{m}>\theta Q_{m}+\varphi Q_{m},\varepsilon \alpha_{m}Q_{m}+\gamma \alpha_{n}Q_{n}+\delta \alpha_{n}Q_{n}>\theta Q_{n}+\varphi Q_{n}$ and considering that the coordinates of the saddle G point must be positive, further analysis demonstrates condition $0<\theta Q_{n}+\varphi Q_{n}-\gamma \alpha_{n}Q_{n}-\delta \alpha_{n}Q_{n}<\varepsilon \alpha_{m}Q_{m},0<\theta Q_{m}+\varphi Q_{m}-\gamma \alpha_{m}Q_{m}-\delta \alpha_{m}Q_{m}<\varepsilon \alpha_{n}Q_{n}$, i.e.,:


$$0<\frac{\theta Q_{n}+\varphi Q_{n}-\gamma \alpha_{n}Q_{n}-\delta \alpha_{n}Q_{n}}{\varepsilon \alpha_{m}Q_{m}}<1$$ (7)

$$0<\frac{\theta Q_{m}+\varphi Q_{m}-\gamma \alpha_{m}Q_{m}-\delta \alpha_{m}Q_{m}}{\varepsilon \alpha_{n}Q_{n}}<1$$ (8)

$$\begin{array}{c} S_{VPGW}\\ =S_{PVG}+S_{VGW}\\ =\frac{\text{1}}{\text{2}}(1-X*)+\frac{\text{1}}{\text{2}}(1-Y*)\\ =1-\frac{\text{1}}{\text{2}}(X*+Y*)\\ =1-\frac{\text{1}}{\text{2}}(\frac{\theta Q_{n}+\varphi Q_{n}-\gamma \alpha_{n}Q_{n}-\delta \alpha_{n}Q_{n}}{\varepsilon \alpha_{m}Q_{m}}+\frac{\theta Q_{m}+\varphi Q_{m}-\gamma \alpha_{m}Q_{m}-\delta \alpha_{m}Q_{m}}{\varepsilon \alpha_{n}Q_{n}}) \end{array}$$ (9)

### 3.1. Quantification of resource stocks

Taking the derivative of $S_{VPGW}$ with respect to $Q_{m}$ yields $SVPGW\prime (Q_{m})=\frac{\text{1}}{\text{2}}(\frac{\theta Q_{n}+\varphi Q_{n}-\gamma \alpha_{n}Q_{n}-\delta \alpha_{n}Q_{n}}{\varepsilon \alpha_{m}Q_{m}}\times \frac{\text{1}}{Q_{m}}+\frac{\theta Q_{m}+\varphi Q_{m}-\gamma \alpha_{m}Q_{m}-\delta \alpha_{m}Q_{m}}{\varepsilon \alpha_{n}Q_{n}}\times \frac{\text{1}}{Q_{m}})$. By combining Equations (7) and (8), it can be inferred that condition $SVPGW\prime (Q_{m})\geq 0$ holds universally, implying that the direction of change in $S_{VPGW}$ aligns with that of Q. Therefore, when the resource quantity Q of either the anchor firm or participating enterprises increases, the probability of the system converging to point V(1,1) rises. Consequently, the likelihood of value creation by both the anchor firm and participating enterprises is enhanced. On the contrary, the main enterprise and participating enterprises are more likely to adopt value acquisition strategies.

### 3.2. Co-creation efficiency coefficient

From Equation (9), it is observed that only the value creation coefficient α appears, while the value capture coefficient β is absent. This implies that *BB* has no impact on the system’s convergence to either point V or O. By taking the derivative of $S_{VPGW}$ with respect to $\alpha_{m}$, we obtain $SVPGW\prime (\alpha_{m})=\frac{\text{1}}{\text{2}}(\frac{\theta Q_{n}+\varphi Q_{n}-\gamma \alpha_{n}Q_{n}-\delta \alpha_{n}Q_{n}}{\varepsilon \alpha_{m}Q_{m}}\times \frac{\text{1}}{\alpha_{m}}+\frac{\gamma Q_{m}+\delta Q_{m}}{\varepsilon \alpha_{n}Q_{n}})$. According to Equation (7), $SVPGW\prime (\alpha_{m})\geq 0$ remains consistently valid, indicating that the direction of change in $S_{VPGW}$ aligns with that of α. When the value creation of the coefficient increases, the probability of the system converging to point V(1,1) rises, and both the anchor firm and participating enterprises are more likely to prioritize value creation. On the contrary, the main enterprise and participating enterprises are more likely to adopt value acquisition strategies.

### 3.3. Co-creation perception coefficient

Within the innovation ecosystem, the shared value co-creation awareness between the anchor firm and participating enterprises enhances the system’s proactiveness in value generation. According to Equation (9), the direction of change in $S_{VPGW}$ aligns with that of γ. Specifically, an increase in the co-creation perception coefficient γ amplifies the probability of the system converging toward point V(1,1), thereby increasing both the anchor firm’s and participants’ likelihood of prioritizing value creation. On the contrary, the main enterprise and participating enterprises are more likely to adopt value acquisition strategies.

### 3.4. Co-creation incentive coefficient

As derived from Equation (9), the trajectory of $S_{VPGW}$ is positively correlated with changes in the co-creation incentive coefficient δ. Specifically, an increase in δ enhances the system’s likelihood of converging to point V(1,1), thereby elevating both the anchor firm’s and participants’ propensity to prioritize value creation strategies. On the contrary, the main enterprise and participating enterprises are more likely to adopt value acquisition strategies.

### 3.5. Co-creation penalty coefficient

As derived from Equation (9), the trajectory of $S_{VPGW}$ is directly proportional to changes in the co-creation penalty coefficient ε. Specifically, an increase in ε amplifies the system’s likelihood of converging to point V(1,1), thereby increasing both the anchor firm’s and participants’ propensity to prioritize value creation strategies. On the contrary, the main enterprise and participating enterprises are more likely to adopt value acquisition strategies.

### 3.6. Co-creation risk coefficient

As derived from Equation (9), the trajectory of $S_{VPGW}$ is **inversely proportional** to changes in the co-creation risk coefficient θ. Specifically, a decrease in θ enhances the system’s likelihood of converging to point V(1,1), thereby increasing both the anchor firm’s and participants’ propensity to prioritize value creation strategies. Conversely, an increase in *RR* shifts the strategic equilibrium toward value capture behaviors, where actors favor expropriating over co-generating value. On the contrary, the main enterprise and participating enterprises are more likely to adopt value acquisition strategies.

### 3.7. Co-creation cost coefficient

As derived from Equation (9), the trajectory of $S_{VPGW}$ is **inversely proportional** to changes in the co-creation cost coefficient ϕ. Specifically, a decrease in ϕ amplifies the system’s likelihood of converging to point V(1,1), thereby increasing both the anchor firm’s and participants’ propensity to prioritize value creation strategies. Conversely, an increase in *RR* shifts the strategic equilibrium toward value capture behaviors, where actors favor expropriating over co-generating value. On the contrary, the main enterprise and participating enterprises are more likely to adopt value acquisition strategies.

## 4. Numerical simulation analysis of evolutionary game dynamics

To further investigate the impact of resource quantities, value co-creation efficacy coefficients, co-creation awareness coefficients, co-creation reward coefficients, co-creation penalty coefficients, co-creation risk coefficients, and co-creation cost coefficients on value co-creation in innovation ecosystems, this study selects key parameters—including co-creation awareness coefficients, reward coefficients, penalty coefficients, risk coefficients, and cost coefficients—for numerical simulation analysis using MATLAB software. The objective is to explore whether the anchor firm and participant firms can actively achieve the ideal state of value co-creation under different parameter configurations.

Grounded in empirical observations of value co-creation between core and participating firms within innovation ecosystems, parameter values for the model are assigned to ensure feasibility and validity. Based on numerical settings from established literature [33] and contextual realities of innovation ecosystems: Resource endowment is set at 500 units for core enterprises and 400 units for participants; Value creation coefficients are specified as 0.3 (core) and 0.7 (participants); Co-creation awareness coefficient = 0.6; Incentive intensity for co-creation = 0.77; Penalty intensity against defection = 0.75; Risk exposure during collaborative processes = 0.34; Implementation cost burden of coordinated efforts = 0.48.

### 4.1. Impact of value co-creation awareness coefficient on collaborative behaviors in innovation ecosystems

**Fig 2:**
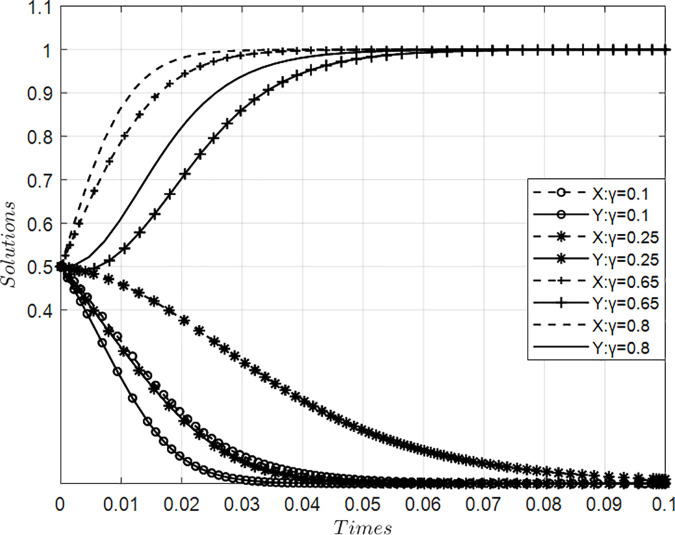
Evolution of value co-creation behaviors with varying co-creation awareness coefficient $\begin{array}{c} \gamma \end{array}$.

Fig 2 presents simulation results illustrating the impact of varying cognition coefficients for value co-creation on behavior dynamics between core and participating firms within an innovation ecosystem, holding other parameters constant. Findings reveal a critical threshold range (0.25–0.65) for co-creation awareness: below this interval, low mutual recognition suppresses willingness to engage in value creation among both actor types, prompting systemic evolution toward value appropriation orientation detrimental to collaborative value generation. Conversely, as cognition exceeds this critical point, increasing at an accelerating rate, initially gradually then sharply converging toward probability = 1, the propensity for both parties to adopt value creation strategies rises substantially. This nonlinear trajectory demonstrates that heightened co-creation awareness drives ecosystem transition toward proactive value creation pathways, thereby enhancing overall innovation efficacy through improved collaborative outcomes.

### 4.2. Impact of value co-creation incentive coefficient on collaborative behaviors among anchor firms in innovation ecosystems

**Fig 3:**
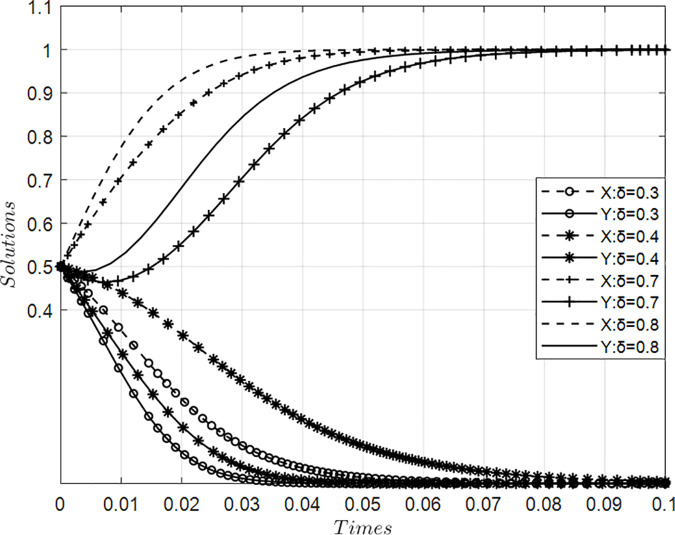
Evolution of value co-creation behaviors with variations in the incentive coefficient $\begin{array}{c} \delta \end{array}$.

Fig 3 presents simulation results illustrating the impact of varying incentive coefficients for value co-creation on behavior dynamics between core and participating firms within an innovation ecosystem, holding other parameters constant. Findings reveal a critical threshold range (0.4–0.7) for reward intensity: below this interval, insufficient motivational stimuli suppress willingness to engage in collaborative value creation among both actor types, prompting systemic evolution toward value appropriation orientation detrimental to synergistic outcomes. Conversely, as incentives exceed this critical point, increasing at an accelerating rate—initially gradually then sharply ascending—the propensity for mutual adoption of value creation strategies rises substantially. This nonlinear trajectory demonstrates that heightened reward mechanisms accelerate transition toward proactive value generation pathways across all innovators, thereby enhancing overall ecosystem efficacy through intensified collaborative engagement.

### 4.3. Impact of value co-creation penalty coefficients on collaborative behaviors among innovation ecosystem agents

**Fig 4:**
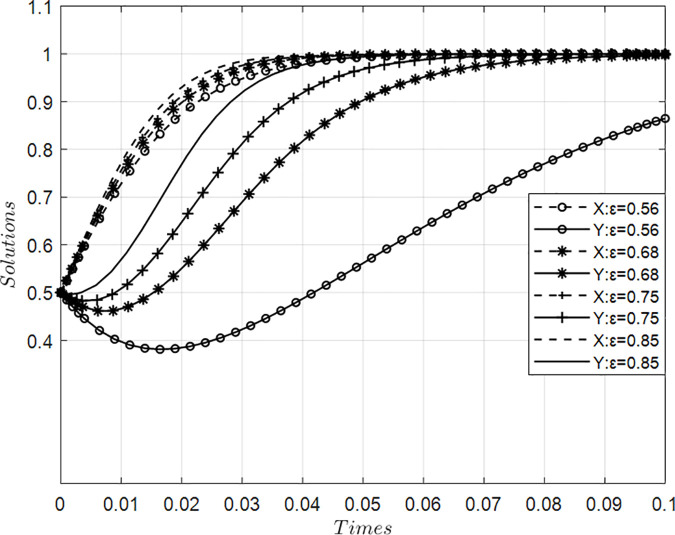
Evolution of value co-creation behaviors under variations in penalty coefficient $\begin{array}{c} \varepsilon \end{array}$.

Fig 4 presents simulation results illustrating the impact of varying penalty coefficients for value co-creation on behavior dynamics between core and participating firms within an innovation ecosystem, holding other parameters constant. Findings demonstrate that as punitive measures intensify progressively—initially gradually then sharply accelerating—the likelihood of both actor types adopting value creation strategies increases substantially. Specifically, evolutionary trajectories exhibit initial gradual ascension followed by steep upward momentum converging toward asymptotic stability near probability = 1. These results indicate that strengthened disciplinary mechanisms significantly accelerate systemic transition toward proactive value generation pathways across all innovators, thereby enhancing overall ecosystem efficacy through enforced collaborative commitment.

### 4.4. Impact of value co-creation risk coefficients on collaborative behaviors among innovation ecosystem agents

**Fig 5:**
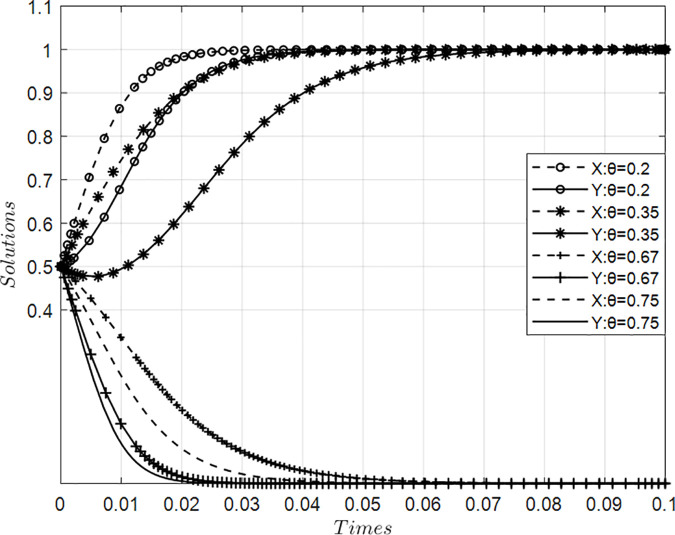
Evolution of value co-creation behaviors under variations in risk coefficient $\begin{array}{c} \theta \end{array}$.

Fig 5 presents simulation results illustrating the impact of varying risk coefficients for value co-creation on behavior dynamics between core and participating firms within an innovation ecosystem, holding other parameters constant. Findings reveal a critical threshold range (0.35–0.67) for collaborative risk exposure: below this interval, progressive reduction in perceived risks accelerates mutual adoption of value creation strategies at an increasingly rapid pace—indicating heightened willingness among both actor types to engage in synergistic activities. Conversely, when systemic risks exceed this critical point, elevated uncertainty suppresses motivation for joint value generation, prompting strategic shifts toward passive value appropriation or defensive resource protection. These dynamics demonstrate that escalating co-creation risks drive ecosystem evolution toward suboptimal pathways characterized by diminished collaborative engagement, thereby constraining overall innovation efficacy through inhibited collective value potential.

### 4.5. Impact of value co-creation cost coefficients on collaborative behaviors among innovation ecosystem agents

**Fig 6:**
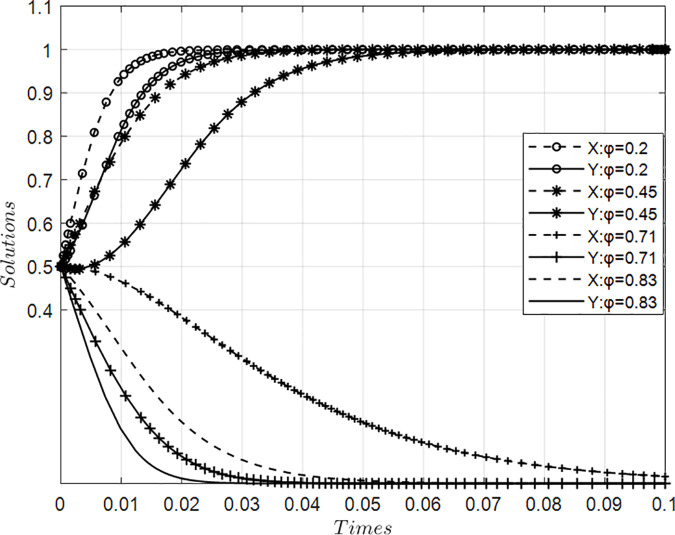
Evolution of value co-creation behaviors under variations in cost coefficient $\begin{array}{c} \phi \end{array}$.

Fig 6 presents simulation results illustrating the impact of varying cost coefficients for value co-creation on behavior dynamics between core and participating firms within an innovation ecosystem, holding other parameters constant. Findings reveal a critical threshold range (0.45–0.71) for collaborative implementation costs: above this interval, excessive burdens suppress willingness to engage in joint value creation among both actor types, driving systemic evolution toward passive appropriation or defensive positioning that undermines collective innovation efficacy. Conversely, as costs decrease below this critical point—with declining marginal expense accelerating mutual adoption of value creation strategies at progressively higher rates—the propensity for synergistic engagement intensifies substantially. This nonlinear trajectory demonstrates that optimized resource allocation efficiency enables transition toward proactive value generation pathways across all participants, thereby enhancing overall ecosystem performance through sustained collaborative investment.

## 5. Research conclusions and prospects

### 5.1. Research conclusions

Grounded in value co-creation theory and bounded rationality of core versus participating firms within innovation ecosystems, this study constructs an evolutionary game model incorporating seven determinants—resource endowment, co-creation efficacy, cognitive orientation toward collaboration, incentive mechanisms, punitive measures, risk exposure, and implementation costs. Using evolutionary game methodology, we analyze how these factors influence value co-creation behaviors, with numerical simulations providing empirical validation of theoretical predictions regarding their dynamic impacts on collaborative innovation processes.

Analysis of the evolutionary game system and numerical simulations reveal three key findings: Frist, increasing resource endowment, value creation coefficients, co-creation awareness coefficients, reward intensity, and penalty severity elevates the probability of convergence to equilibrium point ESS, indicating enhanced stability in value creation strategies for both core and participating firms. Second, reducing risk exposure coefficients and implementation cost burdens similarly increases convergence likelihood toward ESS, facilitating more robust collaborative value generation pathways. Third, value appropriation coefficients exhibit no significant influence on systemic convergence dynamics, implying their irrelevance to cooperative strategy stabilization. Collectively, these results demonstrate that resource availability, value creation mechanisms, cognitive alignment, incentive structures, and disciplinary measures exert positive effects on innovation ecosystem value co-creation, whereas perceived risks and operational costs impose constraining negative impacts on collaborative efficacy.

Based on the findings of this study, we propose the following recommendations for enhancing value co-creation through coordinated value creation between core and participating firms within innovation ecosystems: Firstly, individual enterprises should strengthen their resource advantages by expanding reserves to establish sustainable niche differentiation characterized by robust complementary resource endowments. Secondly, ecosystem actors must cultivate heightened co-creation awareness while refining incentive-penalty frameworks—intensifying rewards for proactive value generation and penalties for opportunistic behaviors to elevate systemic capacity for collaborative innovation. Thirdly, internal value co-creation mechanisms require optimization through periodic assessment of value creation activities, which reduces implementation costs and mitigates associated risks, thereby facilitating balanced value appropriation alongside collective value generation across all participants.

Compared with existing research, this study holds both practical and theoretical significance as follows: First, grounded in the value co-creation perspective, we examine how factors such as resource endowment, co-creation efficacy, cognitive orientation, incentive mechanisms, punitive measures, risk exposure, and implementation costs influence strategic decision-making regarding value co-creation within innovation ecosystems. This provides a foundation for enhancing their capacity to generate collaborative value, thereby offering actionable insights for practitioners. Second, leveraging evolutionary game theory, our analysis explores value co-creation behavior through dual lenses of value creation and appropriation, advancing theoretical understanding by integrating dynamic strategic interactions into innovation ecosystem and value co-creation frameworks.

### 5.2. Research prospects

This study, focusing on value creation and appropriation strategies of core versus participating firms within innovation ecosystems from a value co-creation perspective, offers valuable insights for enhancing collaborative value generation capacity. However, three key limitations warrant attention: First, while internal actor dynamics are prioritized, external factors such as market demand volatility and policy environment shifts—critical determinants of strategic behavior—are not incorporated. Future research could introduce stochastic modeling to examine how these variables shape value co-creation processes. Second, our bilateral framework (core vs. participants) excludes analysis of multi-agent interactions involving governments, intermediaries, or financial institutions; extending this to multi-party game models would clarify how diverse stakeholder logics influence systemic collaboration. Third, although idealized assumptions simplify theoretical exploration, they create divergence from real-world complexity. Subsequent studies should bridge this gap by refining parameterization through empirical data integration, thereby strengthening alignment between theoretical constructs and practical applications.

## Supporting information

S1 File Supplementary materials. Author information.
(DOCX)

S2 File Figshare. Data availability.
(DOCX)
