# Supplementary material for: A study on collaboration innovation: Perspectives of innovative ecosystems of value co-creation using evolutionary game theory
Source: PLoS One. 2026 Feb 11;21(2):e0339295. doi: 10.1371/journal.pone.0339295 (PMC12893565; doi:10.1371/journal.pone.0339295)
Supplement: S1 File — Author information. (DOCX) [file pone.0339295.s001.docx]

**Title：A STUDY ON COLLABORATION INNOVATION: PERSPECTIVES OF INNOVATIVE ECOSYSTEMS OF VALUE CO-CREATION USING EVOLUTIONARY GAME THEORY**

**Author 1:** Shi Xiaowei (1991 -), male, born in Zongyang, Anhui Province, doctoral candidate, majoring in science and technology and innovation management;

**Author 2:** Wang Jifa (1964 -), male, professor, born in Yingkou, Liaoning Province, doctoral supervisor, with research interests in science and technology and innovation management, strategic management, and decision-making technology.

**Corresponding author:** Xiaowei Shi

**Research institutions:** School of Management, Shenyang University of Technology

**Address:** 111 Shenliao West Road, Shenyang Economic Development Zone, Liaoning Province

**Email Id:** ives1113@smail.sut.edu.cn
