# Supplementary material for: A study on collaboration innovation: Perspectives of innovative ecosystems of value co-creation using evolutionary game theory
Source: PLoS One. 2026 Feb 11;21(2):e0339295. doi: 10.1371/journal.pone.0339295 (PMC12893565; doi:10.1371/journal.pone.0339295)
Supplement: S2 File — Data availability. (DOCX) [file pone.0339295.s002.docx]

clc;

clear;

qm=500,qn=400,an=0.3,am=0.7,r=0.1,c=0.77,e=0.75,d=0.34,f=0.48;

set(0,'defaultfigurecolor','w')

%the 1st X,Y

[t,x]=ode45(@(t,x) differential1215(t,x,qm,qn,an,am,r,c,e,d,f),[0,0.1],[0.5,0.5]);

points=1:1:length(t);

figure(1)

plot(t,x(:,1),'ko--','linewidth',1,'markersize',4,'markerindices',points);

hold on

plot(t,x(:,2),'ko-','linewidth',1,'markersize',4,'markerindices',points);

grid on

hold on

set(gca,'XTick',[0:0.01:0.1],'YTick',[0.4:0.1:1.1])

axis([0 0.1 0.1 1.1])

%the 2nd X,Y

qm=500,qn=400,an=0.3,am=0.7,r=0.25,c=0.77,e=0.75,d=0.34,f=0.48;

[t,x]=ode45(@(t,x) differential1215(t,x,qm,qn,an,am,r,c,e,d,f),[0,0.1],[0.5,0.5]);

points=1:1:length(t);

figure(1)

plot(t,x(:,1),'k*--','linewidth',1,'markersize',6,'markerindices',points);

hold on

plot(t,x(:,2),'k*-','linewidth',1,'markerindices',points);

hold on

%the 3nd X,Y

qm=500,qn=400,an=0.3,am=0.7,r=0.65,c=0.77,e=0.75,d=0.34,f=0.48;

[t,x]=ode45(@(t,x) differential1215(t,x,qm,qn,an,am,r,c,e,d,f),[0,0.1],[0.5,0.5]);

points=1:1:length(t);

figure(1)

plot(t,x(:,1),'k+--','linewidth',1,'markersize',4,'markerindices',points);

hold on

plot(t,x(:,2),'k+-','linewidth',1,'markerindices',points);

hold on

%the 4nd X,Y

qm=500,qn=400,an=0.3,am=0.7,r=0.8,c=0.77,e=0.75,d=0.34,f=0.48;

[t,x]=ode45(@(t,x) differential1215(t,x,qm,qn,an,am,r,c,e,d,f),[0,0.1],[0.5,0.5]);

points=1:1:length(t);

figure(1)

plot(t,x(:,1),'k--','linewidth',1,'markersize',4,'markerindices',points);

hold on

plot(t,x(:,2),'k-','linewidth',1,'markersize',4,'markerfacecolor','m','markerindices',points);

hold on

xlabel('$Times$','interpreter','latex');

ylabel('$Solutions$','interpreter','latex');

legend('X:¦Ã=0.1','Y:¦Ã=0.1','X:¦Ã=0.25','Y:¦Ã=0.25','X:¦Ã=0.65','Y:¦Ã=0.65','X:¦Ã=0.8','Y:¦Ã=0.8');

%%%the 2rd image

clc;

clear;

qm=500,qn=400,an=0.3,am=0.7,r=0.6,c=0.3,e=0.75,d=0.34,f=0.48;

set(0,'defaultfigurecolor','w')

%the 1st X,Y

[t,x]=ode45(@(t,x) differential1215(t,x,qm,qn,an,am,r,c,e,d,f),[0,0.1],[0.5,0.5]);

points=1:1:length(t);

figure(2)

plot(t,x(:,1),'ko--','linewidth',1,'markersize',4,'markerindices',points);

hold on

plot(t,x(:,2),'ko-','linewidth',1,'markersize',4,'markerindices',points);

grid on

hold on

set(gca,'XTick',[0:0.01:0.1],'YTick',[0.4:0.1:1.1])

axis([0 0.1 0.1 1.1])

%the 2nd X,Y

qm=500,qn=400,an=0.3,am=0.7,r=0.6,c=0.4,e=0.75,d=0.34,f=0.48;

[t,x]=ode45(@(t,x) differential1215(t,x,qm,qn,an,am,r,c,e,d,f),[0,0.1],[0.5,0.5]);

points=1:1:length(t);

figure(2)

plot(t,x(:,1),'k*--','linewidth',1,'markersize',6,'markerindices',points);

hold on

plot(t,x(:,2),'k*-','linewidth',1,'markerindices',points);

hold on

%the 3nd X,Y

qm=500,qn=400,an=0.3,am=0.7,r=0.6,c=0.7,e=0.75,d=0.34,f=0.48;

[t,x]=ode45(@(t,x) differential1215(t,x,qm,qn,an,am,r,c,e,d,f),[0,0.1],[0.5,0.5]);

points=1:1:length(t);

figure(2)

plot(t,x(:,1),'k+--','linewidth',1,'markersize',4,'markerindices',points);

hold on

plot(t,x(:,2),'k+-','linewidth',1,'markerindices',points);

hold on

%the 4nd X,Y

qm=500,qn=400,an=0.3,am=0.7,r=0.6,c=0.8,e=0.75,d=0.34,f=0.48;

[t,x]=ode45(@(t,x) differential1215(t,x,qm,qn,an,am,r,c,e,d,f),[0,0.1],[0.5,0.5]);

points=1:1:length(t);

figure(2)

plot(t,x(:,1),'k--','linewidth',1,'markersize',4,'markerindices',points);

hold on

plot(t,x(:,2),'k-','linewidth',1,'markersize',4,'markerfacecolor','m','markerindices',points);

hold on

xlabel('$Times$','interpreter','latex');

ylabel('$Solutions$','interpreter','latex');

legend('X:¦Ä=0.3','Y:¦Ä=0.3','X:¦Ä=0.4','Y:¦Ä=0.4','X:¦Ä=0.7','Y:¦Ä=0.7','X:¦Ä=0.8','Y:¦Ä=0.8');

%%%the 3rd image

clc;

clear;

qm=500,qn=400,an=0.3,am=0.7,r=0.6,c=0.77,e=0.56,d=0.34,f=0.48;

set(0,'defaultfigurecolor','w')

%the 1st X,Y

[t,x]=ode45(@(t,x) differential1215(t,x,qm,qn,an,am,r,c,e,d,f),[0,0.1],[0.5,0.5]);

points=1:1:length(t);

figure(3)

plot(t,x(:,1),'ko--','linewidth',1,'markersize',4,'markerindices',points);

hold on

plot(t,x(:,2),'ko-','linewidth',1,'markersize',4,'markerindices',points);

grid on

hold on

set(gca,'XTick',[0:0.01:0.1],'YTick',[0.4:0.1:1.1])

axis([0 0.1 0.1 1.1])

%the 2nd X,Y

qm=500,qn=400,an=0.3,am=0.7,r=0.6,c=0.77,e=0.68,d=0.34,f=0.48;

[t,x]=ode45(@(t,x) differential1215(t,x,qm,qn,an,am,r,c,e,d,f),[0,0.1],[0.5,0.5]);

points=1:1:length(t);

figure(3)

plot(t,x(:,1),'k*--','linewidth',1,'markersize',6,'markerindices',points);

hold on

plot(t,x(:,2),'k*-','linewidth',1,'markerindices',points);

hold on

%the 3nd X,Y

qm=500,qn=400,an=0.3,am=0.7,r=0.6,c=0.77,e=0.75,d=0.34,f=0.48;

[t,x]=ode45(@(t,x) differential1215(t,x,qm,qn,an,am,r,c,e,d,f),[0,0.1],[0.5,0.5]);

points=1:1:length(t);

figure(3)

plot(t,x(:,1),'k+--','linewidth',1,'markersize',4,'markerindices',points);

hold on

plot(t,x(:,2),'k+-','linewidth',1,'markerindices',points);

hold on

%the 4nd X,Y

qm=500,qn=400,an=0.3,am=0.7,r=0.6,c=0.77,e=0.85,d=0.34,f=0.48;

[t,x]=ode45(@(t,x) differential1215(t,x,qm,qn,an,am,r,c,e,d,f),[0,0.1],[0.5,0.5]);

points=1:1:length(t);

figure(3)

plot(t,x(:,1),'k--','linewidth',1,'markersize',4,'markerindices',points);

hold on

plot(t,x(:,2),'k-','linewidth',1,'markersize',4,'markerfacecolor','m','markerindices',points);

hold on

xlabel('$Times$','interpreter','latex');

ylabel('$Solutions$','interpreter','latex');

legend('X:¦Å=0.56','Y:¦Å=0.56','X:¦Å=0.68','Y:¦Å=0.68','X:¦Å=0.75','Y:¦Å=0.75','X:¦Å=0.85','Y:¦Å=0.85');

%%%the 4rd image

clc;

clear;

qm=500,qn=400,an=0.3,am=0.7,r=0.6,c=0.77,e=0.75,d=0.2,f=0.48;

set(0,'defaultfigurecolor','w')

%the 1st X,Y

[t,x]=ode45(@(t,x) differential1215(t,x,qm,qn,an,am,r,c,e,d,f),[0,0.1],[0.5,0.5]);

points=1:1:length(t);

figure(4)

plot(t,x(:,1),'ko--','linewidth',1,'markersize',4,'markerindices',points);

hold on

plot(t,x(:,2),'ko-','linewidth',1,'markersize',4,'markerindices',points);

grid on

hold on

set(gca,'XTick',[0:0.01:0.1],'YTick',[0.4:0.1:1.1])

axis([0 0.1 0.1 1.1])

%the 2nd X,Y

qm=500,qn=400,an=0.3,am=0.7,r=0.6,c=0.77,e=0.75,d=0.35,f=0.48;

[t,x]=ode45(@(t,x) differential1215(t,x,qm,qn,an,am,r,c,e,d,f),[0,0.1],[0.5,0.5]);

points=1:1:length(t);

figure(4)

plot(t,x(:,1),'k*--','linewidth',1,'markersize',6,'markerindices',points);

hold on

plot(t,x(:,2),'k*-','linewidth',1,'markerindices',points);

hold on

%the 3nd X,Y

qm=500,qn=400,an=0.3,am=0.7,r=0.6,c=0.77,e=0.75,d=0.67,f=0.48;

[t,x]=ode45(@(t,x) differential1215(t,x,qm,qn,an,am,r,c,e,d,f),[0,0.1],[0.5,0.5]);

points=1:1:length(t);

figure(4)

plot(t,x(:,1),'k+--','linewidth',1,'markersize',4,'markerindices',points);

hold on

plot(t,x(:,2),'k+-','linewidth',1,'markerindices',points);

hold on

%the 4nd X,Y

qm=500,qn=400,an=0.3,am=0.7,r=0.6,c=0.77,e=0.75,d=0.75,f=0.48;

[t,x]=ode45(@(t,x) differential1215(t,x,qm,qn,an,am,r,c,e,d,f),[0,0.1],[0.5,0.5]);

points=1:1:length(t);

figure(4)

plot(t,x(:,1),'k--','linewidth',1,'markersize',4,'markerindices',points);

hold on

plot(t,x(:,2),'k-','linewidth',1,'markersize',4,'markerfacecolor','m','markerindices',points);

hold on

xlabel('$Times$','interpreter','latex');

ylabel('$Solutions$','interpreter','latex');

legend('X:¦È=0.2','Y:¦È=0.2','X:¦È=0.35','Y:¦È=0.35','X:¦È=0.67','Y:¦È=0.67','X:¦È=0.75','Y:¦È=0.75');

%%%the 5rd image

clc;

clear;

qm=500,qn=400,an=0.3,am=0.7,r=0.6,c=0.77,e=0.75,d=0.34,f=0.2;

set(0,'defaultfigurecolor','w')

%the 1st X,Y

[t,x]=ode45(@(t,x) differential1215(t,x,qm,qn,an,am,r,c,e,d,f),[0,0.1],[0.5,0.5]);

points=1:1:length(t);

figure(5)

plot(t,x(:,1),'ko--','linewidth',1,'markersize',4,'markerindices',points);

hold on

plot(t,x(:,2),'ko-','linewidth',1,'markersize',4,'markerindices',points);

grid on

hold on

set(gca,'XTick',[0:0.01:0.1],'YTick',[0.4:0.1:1.1])

axis([0 0.1 0.1 1.1])

%the 2nd X,Y

qm=500,qn=400,an=0.3,am=0.7,r=0.6,c=0.77,e=0.75,d=0.34,f=0.45;

[t,x]=ode45(@(t,x) differential1215(t,x,qm,qn,an,am,r,c,e,d,f),[0,0.1],[0.5,0.5]);

points=1:1:length(t);

figure(5)

plot(t,x(:,1),'k*--','linewidth',1,'markersize',6,'markerindices',points);

hold on

plot(t,x(:,2),'k*-','linewidth',1,'markerindices',points);

hold on

%the 3nd X,Y

qm=500,qn=400,an=0.3,am=0.7,r=0.6,c=0.77,e=0.75,d=0.34,f=0.71;

[t,x]=ode45(@(t,x) differential1215(t,x,qm,qn,an,am,r,c,e,d,f),[0,0.1],[0.5,0.5]);

points=1:1:length(t);

figure(5)

plot(t,x(:,1),'k+--','linewidth',1,'markersize',4,'markerindices',points);

hold on

plot(t,x(:,2),'k+-','linewidth',1,'markerindices',points);

hold on

%the 4nd X,Y

qm=500,qn=400,an=0.3,am=0.7,r=0.6,c=0.77,e=0.75,d=0.34,f=0.83;

[t,x]=ode45(@(t,x) differential1215(t,x,qm,qn,an,am,r,c,e,d,f),[0,0.1],[0.5,0.5]);

points=1:1:length(t);

figure(5)

plot(t,x(:,1),'k--','linewidth',1,'markersize',4,'markerindices',points);

hold on

plot(t,x(:,2),'k-','linewidth',1,'markersize',4,'markerfacecolor','m','markerindices',points);

hold on

xlabel('$Times$','interpreter','latex');

ylabel('$Solutions$','interpreter','latex');

legend('X:¦Õ=0.2','Y:¦Õ=0.2','X:¦Õ=0.45','Y:¦Õ=0.45','X:¦Õ=0.71','Y:¦Õ=0.71','X:¦Õ=0.83','Y:¦Õ=0.83');
